# Increase of circulating miR-223 and insulin-like growth factor-1 is associated with the pathogenesis of acute ischemic stroke in patients

**DOI:** 10.1186/1471-2377-14-77

**Published:** 2014-04-08

**Authors:** Yang Wang, Yu Zhang, Jun Huang, Xiaoyan Chen, Xiang Gu, Yongting Wang, Lili Zeng, Guo-Yuan Yang

**Affiliations:** 1 Department of neurology, Ruijin Hospital, Shanghai Jiao Tong University, School of Medicine, 197 Ruijin Er Road, 200025 Shanghai, China; 2 Neuroscience and Neuroengineering Research Center, Med-X Research Institute and School of Biomedical Engineering, Shanghai Jiao Tong University, 1954 Hua Shan Road, 200030 Shanghai, China

**Keywords:** Human, Ischemia, microRNA-223, Stroke

## Abstract

**Background:**

The relationship between circulating microRNA-223 and pathogenesis of acute ischemic stroke is unknown. Here we investigated the roles and possible targets of circulating microRNA-223 in human ischemic stroke within the first 72 hours.

**Methods:**

Blood samples were collected from patients within 72 hours after cerebral ischemia (n = 79) and compared with healthy control samples (n = 75). The level of possible downstream factors of microRNA-223 including insulin-like growth factor-1, insulin-like growth factor-1 receptor and interleukin-6 was examined by ELISA assay. The relationship between the microRNA-223 level and NIHSS scores, TOAST subtypes, and infarct volume was analyzed respectively. In addition, twelve adult male CD-1 mice underwent middle cerebral artery occlusion using the suture technique. Circulating blood and brain tissue in the ischemic ipsilateral hemisphere were collected at 24 hours after middle cerebral artery occlusion. microRNA-223 was detected by real-time polymerase chain reactions.

**Results:**

microRNA-223 levels in the circulating blood of acute ischemic stroke patients were greatly increased compared to the control (*p* < 0.05). microRNA-223, which were negatively correlated with NIHSS scores (*r* = −0.531, *p* < 0.01) and infarct volume (*r* = −0.265, *p* = 0.039), was significantly up-regulated in large artery and small artery strokes. The plasma level of insulin-like growth factor-1 was positively associated with that of microRNA-223 (*r* = 0.205, *p* = 0.022). Moreover, microRNA-223 in blood and brain were positively correlated (*r* = 0.834, *p* < 0.05), and they were up-regulated significantly in mice that underwent middle cerebral artery occlusion (*p* < 0.05).

**Conclusions:**

Our results suggest that microRNA-223 is associated with acute ischemic stroke and possibly plays a role in stroke through up-regulating growth factor such as insulin-like growth factor-1 gene.

## Background

microRNAs (miRNAs) are a class of 22 nucleotides, short non-coding RNA molecules conserved in the genomes of animals, plants and viruses [1]. miRNAs are important regulators of the development and function of the brain [2]. Changes of miRNAs are associated with high risk factors of stroke occurrence [3], neuronal cell death [4], excitotoxicity and oxidative damage [5], inflammatory reaction [6], blood–brain barrier disruption and brain edema formation [7,8]. miRNAs profiling is being utilized to identify subtypes [9], diagnose diseases [10], instruct treatment plans [11] and predict long term outcomes [12].

Great attention has been paid to miR-223 because it can regulate cell cycle, tumor invasiveness, haematopoietic differentiation and immune cell function [13]. miR-223 is located in the X chromosome. Some known transcriptional factors including NFI-A and C/EBPα regulate miR-223 expression [14]. Studies demonstrated that miR-223 is mainly expressed in bone marrow and plays an important role in granulopoiesis [15]. miR-223 overexpression down-regulates interleukin-6 (IL-6) and IL-1β expression in TLR-activated macrophages [16]. During differentiation or tumor progression, miR-223 suppresses cell proliferation by targeting insulin-like growth factor-1 receptor (IGF1R) [17]. In hepatic ischemia/reperfusion injury, miR-223 is greatly up-regulated and positively correlates with serum markers of ischemic injury [18]. In the central nervous system miR-223 is also highly present and is neuroprotective by targeting GluR2 and NR2B subunits of the glutamate receptor [19].

However, the function of miR-223 associated with acute ischemic brain injury (less than 72 hours) remains unknown. The changes of miR-223 levels for stroke diagnosis and their relationship with clinical confound factors need to be explored. Here, we analyzed changes of miR-223 levels in acute ischemic stroke patients and animal ischemia model. We then assessed the relationship between the alteration of circulating miR-223 levels and the NIHSS scores, subtypes and infarct volume of acute stroke patients. To explore the regulatory mechanism of miR-223 in the ischemic pathological process, we also studied the down-stream insulin-like growth factor-1 (IGF-1), IGF1R and IL-6 changes in stroke patients.

## Methods

### Study subject

Subjects were enrolled in the study from July 2012 to January 2013 and were approved by the Institutional Review Board (IRB) of the Shanghai Jiao Tong University, and gave informed consent. The control group included subjects whose age and gender matched that of the stroke patients. Demographic changes, associated laboratory inspection, imaging information, which included blood pressure, fasting blood glucose, cholesterol, triglyceride, CT, MRI, MR angiography, carotid artery ultrasonography and cardiac ultrasonography were also collected to analyze. Whether subjects taking medicines including antidiabets, hypotensor or platelet aggregation drugs was also recorded. The exclusion criteria included recurrent stroke, intracranial tumor, multiple trauma, hematological system diseases, renal or liver failure, acute infectious diseases and other diseases affecting the hemogram. If the time from the onset of stroke symptoms to blood sample collection was longer than 72 hours, the patient was excluded.

The severity of ischemic stroke was assessed by the National Institutes of Health Stroke Scale (NIHSS) [20]. The patients were classified into the following groups: large-artery atherosclerosis (LA, n = 37), cardioembolism (CE, n = 5), small artery stroke (SA, n = 9) and undetermined etiology (UN, n = 28) by the Trial of Org 10172 in Acute Stroke Treatment (TOAST) [21]. The infarct volumes were calculated by ABC/2 method [22]. The risk factors were defined as following: hypertension: blood pressure above 140/90 mmHg; hyperlipidemia: total cholesterol level ≥ 0.7 mmol/L, triglyceride level ≥ 1.8 mmol/L and HDL level < 1 mmol/L; Diabetes mellitus: fasting-blood glucose level > 6.1 mmol/L or HbA1c ≥ 7% [23].

### Middle cerebral artery occlusion in mice

Procedures for the use of laboratory animals were approved by the Shanghai Jiao Tong University Institutional Animal Care and Use Committee, Shanghai, China. Adult male CD-1 mice (n = 12) weighing 25–30 grams were anesthetized with ketamine/xylazine (100 mg/10 mg/kg, Sigma, San Louis, MO). Body temperature was controlled and maintained at 37 ± 0.3°C using a heating pad (RWD Life Science, Shenzhen, China) during the anesthesia period. After isolation of left common carotid artery (CCA), external and internal carotid artery (ECA, ICA), a silicone-coated 6–0 suture (Covidien, Mansfield, MA) was gently inserted from the ECA stump to the ICA, and stopped at the opening of the MCA. The distance from the bifurcation of ICA/ECA to MCA was 10 ± 0.5 mm [24]. Successful occlusion was verified by a laser Doppler flowmeter (Moor Instruments, Devon, UK). Mice with surface cerebral blood flow that was more than 15% of baseline were excluded from the experiment. Sham-operated mice underwent the same surgery procedure except inserting the suture into the ICA.

### Preparation of blood and brain samples

A 4 ml blood sample from acute ischemic stroke patients was collected into tubes containing ethylenediaminetetraacetic acid, and centrifuged at 1500 g for 10 minutes at room temperature. Then the erythrocytes were dissociated with erythrocyte lysing solution and discarded, the remaining leucocytes were held for total RNA extraction.

The brains of mice were rapidly removed and cut into four coronal sections 2 mm apart. The second slice was divided into ischemic ipsilateral and contralateral hemispheres. The ischemic ipsilateral brain tissue and blood collected for total RNA extraction using TRIzol and TRIzol LS reagent respectively (Invitrogen, Carlsbad).

### Reverse transcription and Real-time PCR

RNA concentration and purity were detected by the NanoDrop1000 spectrophotometer (Thermo, Wilmington, DE). The samples of absorbance at 260–280 nm between 1.8 and 2.0 were adopted. First strand cDNA was synthesized from 10 ng RNA using universal cDNA synthesis kit (EXIQON, Vedbaek, Denmark). The amplification was performed by a fast real-time PCR system (7900 HT, ABI, Foster City, CA) using a SYBR Green master mix (EXQION) following the cycling conditions: 95°C for 10 minutes followed by 40 cycles of 95°C for 10 seconds, and 60°C for 1 minute. miR-223 relative expression was normalized to the endogenous control U6 expression in triplicate and was calculated by the 2^-Δct^ method.

### Enzyme-linked Immunosorbent Assay (ELISA)

The levels of human plasma IGF-1, IGF1R and IL-6 were measured by ELISA kits (Westang Bio-Tech, Shanghai, China) according to the manufacturer’s protocols. The intra- and inter-assay CV are less 10%.

### Statistical analysis

The results were expressed as percentages for categorical variables and as mean ± SD or median and range (25th and 75th percentiles) for the continuous variables depending on whether their distribution was normal or not. The Kolmogorov-Smirnov test was used for testing the normality of the distribution. Proportions were compared using the chi-square test, while the continuous variables between groups were compared with the Student’s t or the Mann–Whitney tests. Spearman’s analysis was used for bivariate correlations depending on their non-normal distribution. ANOVA should be used for comparison among several quantitative variables. The influence of miR-223 levels on a categorical variable was assessed by logistic regression analysis using forward stepwise selection procedures after adjusting for those variables with a proven biological relevance for stoke mobidity to avoid the possibility of finding some spurious associations. Results were expressed as adjusted odds ratios (OR) with the corresponding 95% confidence intervals (95% CI). Significance was set at a probability *p* < 0.05.

## Results

### Baseline clinical characteristics

The characteristics of ischemic stroke patients and controls enrolled in this study are shown in Table 1. On admission, the ages of the stroke patients were 65.1 ± 10.0 years and the ages of the control group were 62.5 ± 6.3 years (*p* = 0.053). The number of males in both groups was about double that of females (male/female = 58/21 in ischemic group and male/female = 50/25 in control group, *p* = 0.383). To eliminate unmatched factor impaction (hypertension, diabetes and hyperlipidemia) on miR-223 expression levels between two groups, a logistic regression was performed. The results of logistic regression suggested that the level of blood miR-223 was a stroke risk factor (*p* = 0.011, adjusted odd ratio = 1.002, 95% CI: 1.000-1.004).

**Table 1:** Clinical characteristics of the healthy control and acute ischemic stroke patients

	**Control**	**Acute ischemic stroke**				***p***
		**1d**	**2d**	**3d**	**Total**	
Total (N)	75	15	22	42	79	1.00
Race (Asian,%)	100%	100%	100%	100%	100%	1.00
Ethnicity (Han,%)	100%	100%	100%	100%	100%	1.00
Age (years, Mean ± SD)	62.5 ± 6.3	70.0 ± 13.46	66.50 ± 9.55	63.0 ± 9.09	65.1 ± 10.0	0.053
Sex (male/female, N)	50/25	11/4	18/4	29/13	58/21	0.383
Hypertension (N,%)	27 (36%)	12 (80%)	17 (77.3%)	32 (76.2%)	61 (77.2%)	<0.05
Diabetes (N,%)	4 (5.3%)	4 (26.7%)	8 (36.4%)	19 (45.2%)	31 (39.2%)	<0.05
Hyperlipidemia (N,%)	28 (37.3%)	10 (66.7%)	14 (63.6%)	24 (57.1%)	48 (60.8%)	<0.05
Cardiopathy (N,%)	2 (2.7%)	4 (26.7%)	1 (4.5%)	2 (4.8%)	7 (8.9%)	0.10
NIHSS (Mean, Min, Max)	NA	4.73 (1, 11)	5.05 (0, 20)	3.10 (0, 10)	3.95 (0, 20)	NA
TS						
LA (N,%)	NA	6 (40%)	14 (63.6%)	17 (40.5%)	37	NA
CE (N,%)	NA	3 (20%)	0	2 (4.8%)	5	NA
SA (N,%)	NA	1 (6.7%)	1 (4.5%)	7 (16.7%)	9	NA
UN (N,%)	NA	5 (33.3%)	7 (31.8%)	16 (38.1%)	28	NA

TS = TOAST subtype; NA, data not available.

### The expression levels and the time course of blood miR-223 in acute ischemic stroke patients

**Figure 1:**
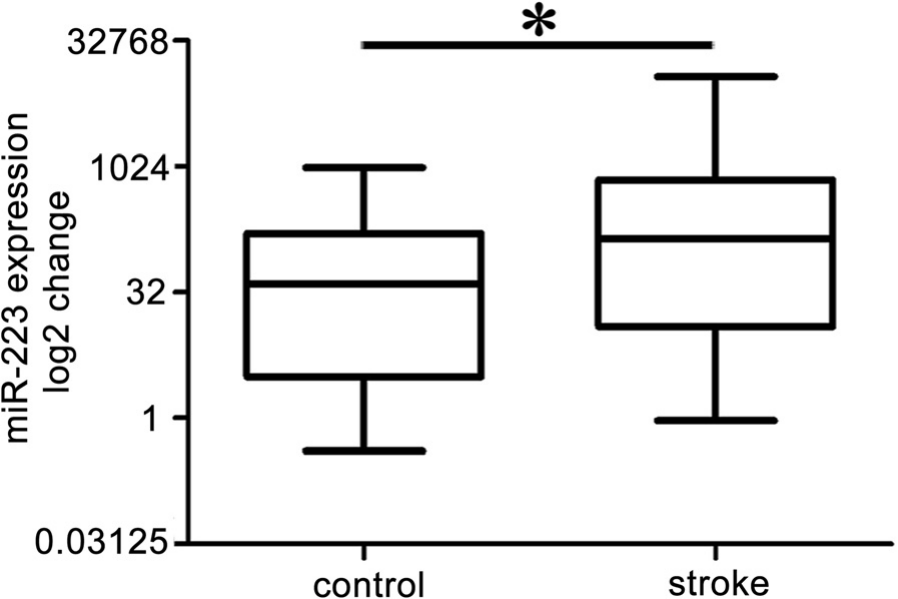
**Blood miR-223 levels were significantly increased in acute ischemic stroke.** Box plots showed the expression of blood miR-223 in patients and control. The Y axis indicated miR-223 expression levels by log2 change. *, *p* < 0.05, stroke patients vs. control. Data are median (25% percentile, 75% percentile), n = 79 in stroke group, n = 75 in control group.

**Figure 2:**
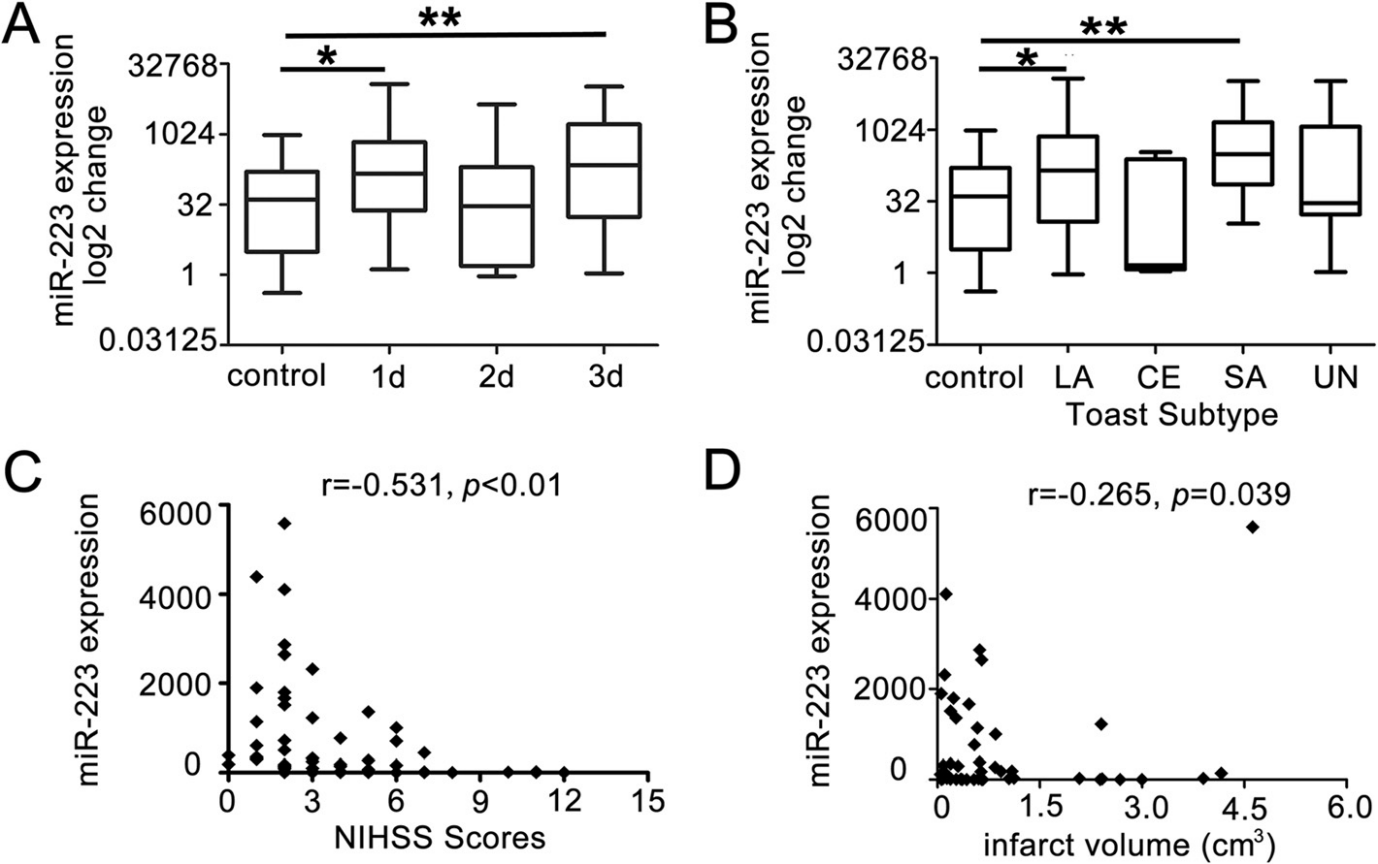
**The time course of miR-223 expression and the correlation analysis between miR-223 and clinical information. A**. miR-223 was highly expressed in 1 day and 3 days stroke patients. Box plots exhibited the time course of miR-223 expression in stroke patients. *, *p* < 0.05, 1 day vs. control; **, *p* < 0.01, 3 days vs. control. n = 75 in control group, n = 15 in 1 day group, n = 22 in 2 days group, n = 42 in 3 days group. **B**. miR-223 was significantly up-regulated in patients with LA and SA strokes. Box plots exhibited the miR-223 expression level among different subtypes. *, *p* < 0.05, LA vs. control; **, *p* < 0.01, SA vs. control. n = 75 in control, n = 37 in LA, n = 5 in CE, n = 9 in SA, n = 28 in UN. Data are median (25% percentile, 75% percentile). The Y axis showed the miR-223 expression levels by log2 change. **C**. The scatterplot showed miR-223 levels were negatively associated with NIHSS scores. *r* = −0.531, *p* < 0.01. **D**. The scatterplot showed miR-223 levels have a negative correlation with infarct volume of stroke patients. *r* = −0.265, *p* = 0.039.

miR-223 levels in the blood of acute ischemic patients were elevated compared with the control subjects [Figure 1, stroke vs. control, 141.57 (12.45, 715.12) vs. 40.64 (3.13, 162.80)]. miR-223 expression was up-regulated in the 1 day and 3 days groups following stroke occurrence compared to the control [Figure 2A, 1 day vs. control, 146.92 (23.72, 700.05) vs. 40.64 (3.13, 162.80), *p* < 0.05; 3 days vs. control, 224.76 (17.41, 1700.63) vs. 40.64 (3.13, 162.80), *p* < 0.01]. In addition, the difference between patient with/without antidiabets, hypotensor or platelet aggregation drugs was analyzed respectively. We did not find the significant difference of the expression levels of miR-223 (data not shown here).

### The relationship between miR-223 and TOAST subtypes, NIHSS scores and infarct volume

miR-223 expression levels of LA or SA stroke patients were significantly increased [Figure 2B, LA vs. control, 143.46 (11.89, 741.23) vs. 40.64 (3.13, 162.80), *p* < 0.05; SA vs. control, 312.35 (73.16, 1466.71) vs. 40.64 (3.13, 162.80), *p* < 0.01]. miR-223 expression levels were negatively associated with NIHSS scores (Figure 2C, *r* = −0.531, *p* < 0.01) and infarct volume (Figure 2D, *r* = −0.265, *p* = 0.039).

### Brain and blood miR-223 in ischemic mice

**Figure 3:**
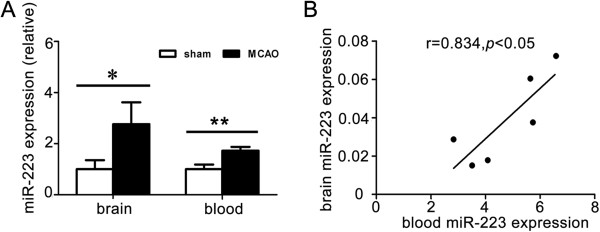
**Blood and brain miR-223 levels were elevated and they have a positive correlation in mice. A**. Bar graphs showed the expression of miR-223 in brain tissue and blood of MCAO mice at 1 day. *, *p* < 0.05; **, *p* < 0.01, MCAO vs. sham. Data are mean ± SD, n = 6 in each group. **B**. The scatter plot demonstrated the positive correlation between blood miR-223 and brain miR-223 at 1 day after ischemia. *r* = 0.834, *p* < 0.05, n = 6.

miR-223 was significantly up-regulated in both brain and blood samples compared to that of the sham in MCAO mice (Figure 3A, brain, MCAO vs. sham, 0.06 ± 0.01 vs. 0.02 ± 0.007, *p* < 0.05; blood, MCAO vs. sham, 5.95 ± 0.92 vs. 3.47 ± 0.43, *p* < 0.01). A positive correlation between the blood miR-223 and the brain miR-223 levels was analyzed by Spearman’s correlation analysis (Figure 3B, *r* = 0.834, *p* < 0.05).

### The correlation between miR-223 and potential target genes

**Figure 4:**
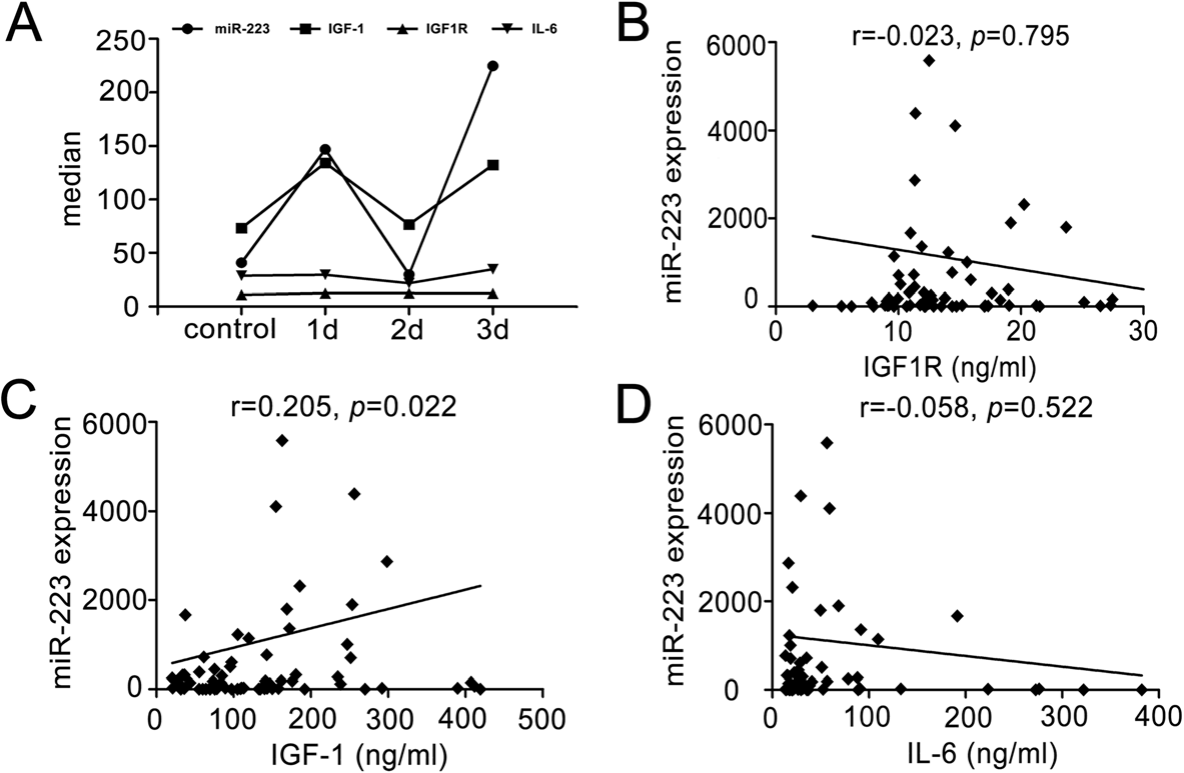
**The relationship between miR-223 and IGF1R, IGF-1 and IL-6. A**. Line chart exhibited the changes of miR-223, IGF-1, IGF1R and IL-6 within 72 hours. Data are median, n = 75 in control, n = 15 in 1 day group, n = 22 in 2 days group, n = 42 in 3 days group. **B**. The scatterplot showed miR-223 was not associated with IGF1R after ischemia. *r* = −0.023, *p* = 0.795. **C**. The scatterplot demonstrated miR-223 was positively correlated to IGF-1. *r* = 0.205, *p* = 0.022. **D**. The scatterplot showed miR-223 was not associated with IL-6 after ischemia. *r* = −0.058, *p* = 0.522.

The levels of plasma IGF-1 and IGF1R were greatly increased in the stroke patients group compared to the control group (Table 2). IGF-1 levels changed with miR-223 levels in the 1 day, 2 days and 3 days groups after ischemia (Figure 4A) and miR-223 expression was positively associated with IGF-1 levels (Figure 4C, *r* = 0.205, *p* = 0.022). There was no significant correlation between miR-223 expression and IGF1R (Figure 4B, *r* = −0.023, *p* = 0.795) and IL-6 (Figure 4D, *r* = −0.058, *p* = 0.522).

**Table 2:** The expression of IGF-1, IGF1R and IL-6 in ischemic stroke patients and control

	**Control N = 75**	**1d N = 15**	**2d N = 22**	**3d N = 42**	**Total N = 79**	***P***
IGF-1 (ng/ml)	73.30 (44.22, 114.54)	134.21 (69.34, 251.37)	76.55 (56.24, 142.51)	132.36 (76.58, 188.79)	111.44 (69.54, 184.22)	0.001
IGF1R (ng/ml)	10.93 (9.87, 12.36)	12.49 (11.33, 17.03)	12.45 (9.34, 15.09)	12.20 (10.07, 17.91)	12.45 (10.31, 16.75)	0.004
IL-6 (ng/ml)	28.56 (19.87, 52.85)	29.52 (22.36, 40.90)	21.57 (19.04, 30.49)	34.73 (21.61, 57.80)	29.04 (20.13, 54.36)	0.909

Data are median (25% quartile, 75% quartile).

## Discussion

In this study, we demonstrated that miR-223 was hyper-expressed in the circulating blood of acute ischemic stroke patients. The severity of stroke and the infarct volume of stroke patients were negatively associated with miR-223 expression. miR-223 levels were greatly increased in patients with LA and SA stroke. The plasma level of IGF-1 has a positive correlation to miR-223 level. Brain miR-223 and blood miR-223 level were significantly elevated and have a positive correlation in MCAO mice.

Previous animals’ experiments demonstrated that miR-223 level was significantly elevated at 24 and 72 hour reperfusion in rat transient MCAO [25] and at 3 and 24 hours in ischemic preconditioning [26]. Another study of miR-223 expression proved that miR-223 was greatly increased in the blood of young stroke patients aged 18–49 years [23]. In our study, we found the similar changes of miR-223 expression in the blood of acute ischemic stroke patients and that the brain miR-223 level positively correlated with blood miR-223 level in ischemic mice. The result suggested that miR-223 is involved in the process of ischemia and hypoxia and implied that blood miR-223 represents the changes of miR-223 in brain response to ischemic stroke.

Blood samples were obtained within 72 hours of stroke onset and molecular studies have shown considerable variability in the acute period (Figure 2A). Maybe this is a relative broad time range for the expression of miR-223. Besides, to obtain a temporal expression profile of miR-223 in patients, it is perfect for the extraction of basal samples from the same patients at 24, 48 and 72 hours. This problem could be solved in further studies. In general, ischemic stroke classified as LA, CE, SA and Undetermined according TOAST subtype classification. We classified inpatients as LA, CE, SA and UN in our study group and found that LA and UN are the main subtypes. This may be different from other studies, we consider it may be caused by the ethnic group. In the study, miR-223 expression of the stroke patients with LA or SA showed similar up-regulation. However, miR-223 showed opposite expression patterns in LA or SA stroke within 6–18 months [23]. The difference might be caused by the different blood sample collection times. NIHSS scores indicate the severity of ischemic stroke. The mean NIHSS in our studies is relative low, suggesting miR-223 is sensitive for mild stroke. Whether miR-223 associated with the severe stroke needs to be further studied. NIHSS scores had a negative correlation with miR-223 expression in our study, implying the potential neuroprotective role of miR-223 in stroke.

The therapeutic potential of IGF-1 for ischemic brain injury had been proved [27,28]. In the study, IGF-1 level changed with miR-223 levels in the 1 day, 2 days and 3 days groups after ischemia, suggesting the modulation of miR-223 on IGF-1 expression. In vitro studies have demonstrated that miR-223 could specifically regulate IGF1R expression [17]. However, we did not find a similar correlation between miR-223 and IGF1R in the blood of patients with acute ischemia. The reason for the difference between in vitro and in vivo studies was unclear. Increasing evidences suggest miR-223 plays a vital role in modulating inflammatory reactions [15]. IL-6 could induce the decrease of miR-223 expression after lipopolysaccharide stimulation in macrophages [16]. But we did not find a correlation between miR-223 and IL-6 levels in vivo. It was plausible to assume that miR-223 takes part in the early inflammatory reaction after stoke through other pathways.

Stroke is a neurological emergency where time has an extraordinary value for clinical or therapeutic decisions. As a result, a useful biomarker for stroke should be determined in a very short period of time. To develop a novel technique quickly detect the changes of miR-223 will greatly help to the diagnosis of stroke. Moreover, RNA stabilizing agent is better than EDTA tube, which is modified in future study.

## Conclusions

The study reported circulating miR-223 based markers for acute ischemic stroke occurrence, subtypes and infarct volume. Our findings suggested IGF-1 might be a new target of miR-223. Further studies with larger sample sizes are needed to assess the clinical application of miR-223 signatures. We believe that miR-223 has great potential as a novel therapeutic target for ischemic stroke.

## Abbreviations

ECA: External carotid artery; IGF-1: Insulin-like growth factor-1; IGF1R: Insulin-like growth factor-1 receptor; IL-1β: interlukin-1β; IL-6: Interlukin-6; ICA: Internal carotid artery; MCAO: middle cerebral artery occlusion; miR-223: microRNA-223; NIHSS: National Institutes of Health Stroke Scale; TOAST: Trial of Org 10172 in acute stroke treatment criteria.

## Competing interests

The authors declare that they have no competing interests.

## Authors’ contributions

YW and YZ were involved in research design, clinical samples and materials collection and experimental performance, analyzing the data as well as drafting the manuscript. JH did animal surgery. XC and XG gave technical assistant. YW discussed the results and edited the part of manuscript. LZ and GYY are the corresponding authors, they took the whole consideration for this study, including research design, data analysis, results discussion, and manuscript preparation. All authors read and approved the final manuscript.

## Pre-publication history

The pre-publication history for this paper can be accessed here:

http://www.biomedcentral.com/1471-2377/14/77/prepub
